# A Vaginitis Classification Method Based on Multi-Spectral Image Feature Fusion

**DOI:** 10.3390/s22031132

**Published:** 2022-02-02

**Authors:** Kongya Zhao, Peng Gao, Sunxiangyu Liu, Ying Wang, Guitao Li, Youzheng Wang

**Affiliations:** 1 School of Aerospace Engineering, Tsinghua University, Beijing 100084, China; zhaoky15@mails.tsinghua.edu.cn (K.Z.); lsxy14@mails.tsinghua.edu.cn (S.L.); ligt@mail.tsinghua.edu.cn (G.L.); yzhwang@mail.tsinghua.edu.cn (Y.W.); 2 Beijing National Research Center for Information Science and Technology, Tsinghua University, Beijing 100084, China; 3 School of Mechatronical Engineering, Beijing Institute Of Technology, Beijing 100081, China; 4 Department of Obstetrics and Gynecology, Beijing Tsinghua Changgung Hospital, School of Clinical Medicine, Tsinghua University, Beijing 102218, China; wya01072@btch.edu.cn

**Keywords:** vaginitis, multi-spectral image, image classification

## Abstract

Vaginitis is one of the commonly encountered diseases of female reproductive tract infections. The clinical diagnosis mainly relies on manual observation under a microscope. There has been some investigation on the classification of vaginitis diseases based on computer-aided diagnosis to reduce the workload of clinical laboratory staff. However, the studies only using RGB images limit the development of vaginitis diagnosis. Through multi-spectral technology, we propose a vaginitis classification algorithm based on multi-spectral image feature layer fusion. Compared with the traditional RGB image, our approach improves the classification accuracy by 11.39%, precision by 15.82%, and recall by 27.25%. Meanwhile, we prove that the level of influence of each spectrum on the disease is distinctive, and the subdivided spectral image is more conducive to the image analysis of vaginitis disease.

## 1. Introduction

Vaginitis is the most common disease of female reproductive tract infections. It is reported [1] that in 2019, about 14.7 million female patients among 20–64 years old have vaginitis in China. As to pathogeny, vaginitis is a general name of various inflammatory diseases of vaginal mucosa caused by different reasons, mainly including aerobic vaginitis (AV), bacterial vaginosis (BV), vulvovaginal candidiasis (VVC), and trichomonas vaginitis (TV). A common disease does not mean it is harmless. On the contrary, it is the cause of serious consequences, such as HPV infection leading to cervical cancer [2,3], miscarriage, premature rupture of membranes, and premature delivery for pregnancy [4]. So, if vaginitis is not treated in time, it will seriously endanger women’s health. However, since vaginitis is caused by a variety of pathogens, its diagnosis and pathogen confirmation is critical before a gynecologist can give the right treatment.

The present diagnosis method for vaginitis highly relies on experienced laboratorians. They observe vagina secreted samples with a microscope and give diagnosis results by their experience. However, for the first hand, after undergoing such manual inspection for a long time, it is very possible for the laboratorians to get tired and thus raise the rate of diagnosis mistakes [5,6,7], especially for large hospitals with more patients. On the other hand, training an experienced laboratorian also requires a long time and high expense, which is less feasible for undeveloped countries. Therefore, computer-aided diagnosis (CAD) is an appropriate way to help inspectors reduce workload and keep a high correctness rate in diagnosis.

Most of the recent studies on CAD for vaginitis are based on support vector machine (SVM), deep learning, and laws texture energy algorithm. The recent literatures are listed in the Table 1. In terms of the SVM algorithm, Song Youyi et al. [8] proposed an automatic detection method for vaginal bacteria based on superpixels and SVM on the Gram-stained vaginal microscopic image; Guo Rui [9] used a convolutional neural network (CNN) to extract features and then used an SVM model to recognize candida on 529 microscope images of leucorrhea. In terms of deep learning algorithms, Zhang Liwei [10] applied a backpropagation (BP) neural network algorithm to identify 86 micro-images of white blood cells; Qin Feiwei et al. [11] proposed a fine-grained white blood cell classification method for microscopic images based on deep residual learning theory and medical field knowledge; Yan Sineng [12] used the Faster R-CNN network as the detection network to detect eight types of targets in gynecological microscopic images. The average recall rate of detection is 74.15%, the average precision rate is 69.94%, and the mAP is 61.74%. Wang Zhongxiao et al. [13] developed a CNN model and evaluated its ability to automatically identify and classify three categories of Nugent scores from microscope images. Regarding the Laws texture energy algorithm, Guo Yukun et al. [14] proposed a fast and effective algorithm to detect and count the number of lactobacilli using the Laws texture energy method; Ma Liwen [15] proposed a method to detect cell texture based on multi-scale Laws texture energy and segmented each component in 1500 microscopic images when recognizing cue cells and epithelial cells. In all, those methods investigate and prove the feasibility of using microscope images as input to diagnose vaginitis.

Limited by technique development, all the previous studies only use RGB images as their input. As we know, an RGB image can be roughly seen as a record of three different spectrum information. Following the line of thinking, if the light from the observed object is divided into multiple disjoint narrow spectrums and each spectrum is recorded as an image respectively, the fined-grained response information of the target to the spectrum is retrieved. This is the basic idea of the multi-spectral imaging technique used in agriculture, military, and environmental monitoring [16]. It is also reported [17,18,19] that the multi-spectral imaging technique achieves higher accuracy or better specificity for disease classification than RGB images.

Built on the previous research on CAD and multi-spectral imaging, the present work proposes a vaginitis classification method based on multi-spectral imaging and feature fusion. The method, named MIDV (Multi-spectral Imaging-based Diagnosis for Vaginitis), is consisted of of three successive parts: single-spectrum feature extraction, multi-spectrum feature fusion, and classifier. The single-spectrum feature extraction part employs a CNN structure to extract the features from the images, each of which corresponds to a different spectrum. The classifier part uses SVM to classify the fused multi-spectrum image feature. Compared with the traditional RGB image, MIDV increases the classification accuracy by 11.39%, precision by 15.82%, and recall rate by 27.25%. Furthermore, it is found that each kind of infection in vaginitis has a unique sensitive spectral band. Intuitively, it means that the features of one infection are more distinctive under a unique spectral band than under others.

The contributions of this paper can be summarized as follows.

- This paper is the first try to introduce a multi-spectral imaging method for the vaginitis diagnosis;
- For the first time, it is found that each kind of vaginitis has a unique sensitive spectral band;
- A classification approach MIDV is designed, which combines deep learning with multi-spectral image feature fusion in the vaginitis domain.

The rest of the paper is organized as follows. Section 2 contains related work, Section 3 is background knowledge, Section 4 is our methodology, Section 5 is the experiment and results, and Section 6 is the conclusions.

## 2. Related Work

### 2.1. Medical Image Analysis Using Transfer Learning Strategy

Transfer learning, which refers to applying knowledge or patterns learned in one specific field or task to another related but different area or problem, has been considered as an effective strategy in deep learning algorithms, especially under the scenario of insufficient data [20]. Due to the high cost of annotation, the medical images dataset is always relatively small, so a transfer learning strategy is appropriate for processing this kind of image. The typical transfer learning procedure in medical image analysis is always like this: first, the CNN model is pre-trained using large non-medical datasets (such as ImageNet), then the convolutional layer of the model is fine-tuned or frozen (that is, the parameters are unchanged), and finally, the fully connected layer is retrained by using a small amount of medical data.

Maghdid Halgurd S et al. [21] carried out COVID-19 detection tasks based on a CNN model pre-trained by natural image. After migration, the accuracy of detection reached as high as 98%; Liu Weixiao et al. [22] proposed an integrated network structure using three natural images pre-training. The trained VGG model there is used as a feature extractor. Multi-scale feature stitching is performed, and the classification AUC is 87.5%; Andre Esteva et al. [23] used GoogleNet Inception v3 architecture to pre-train on ImageNet and then fine-tuned on their dataset. The classification accuracy has reached the level of professional dermatologists; Noorul Wahab et al. [24] used natural images to pre-train ResNet and then used it to detect cell mitosis. The experimental results show that the training method based on transfer learning provides an excellent initial weight, and the training time is reduced, too. Based on those previous research studies, the Inception v3 architecture is chosen by present work.

### 2.2. Multi-Spectral Data Fusion

Data fusion methods [25] in the area of multi-sensor are generally carried out on the layers of data, feature, and decision. Data layer fusion is carried out directly on the collected original data layer. Data synthesis and analysis are conducted before the original data of various sensors are preprocessed. The feature layer fusion refers to the middle layer fusion. It extracts the original information from the sensor before analyzing the feature comprehensively. Decision fusion processes the data of each sensor to make a judgment. and then merge all decisions into one result. The advantage of feature layer fusion lies in realizing considerable information compression, which is conducive to real-time processing. Moreover, because the extracted features are directly related to decision analysis, the fusion results can provide the feature information needed for decision analysis to the maximum extent. Therefore, feature layer fusion is adopted in our algorithm, while data layer fusion and decision layer fusion are used for comparison.

With the popularity of deep learning, more and more data fusion techniques and deep learning models are combined [26,27]. Liu Yu et al. [28] proposed a multi-scale data fusion framework for bone age assessment of X-ray images based on Non-Subsampled Contourlet Transform (NSCT) and CNN. Under this framework, a regression model based on feature-level fusion and a classification model based on decision-level fusion are proposed. The model integrates multiple VGGNet-16 convolutional neural networks to perform further feature extraction on the features decomposed by NSCT so that the description is more precise. Zhang Li et al. [29] proposed a ball screw degradation detection and identification method based on multi-sensor data fusion and Deep Belief Network (DBN). The time-domain signal is converted into the corresponding frequency domain signal and fused by parallel superposition. Then, the fusion result is used as input to train the DBN through unsupervised learning; finally, the softmax classifier is used for classification. Compared with the DBN method using unfused datasets, the experimental results show that this method has better accuracy and stability on the training set and test set. Fu Huiyuan et al. [30] proposed a multi-scale feature fusion convolutional neural network (MCFF-CNN) based on the residual network for vehicle color recognition. MCFF-CNN realizes the multi-scale fusion of image features by combining the output features of different network layers while fusing the output features of the deep network and the shallow network to obtain deeper features of the vehicle image. This method can recognize the color of vehicles under different light conditions, and it has good robustness.

## 3. Background Knowledge

### 3.1. Inception v3

CNN (Convolutional Neural Networks) is the most popular deep learning framework, and it has been widely adopted in the task of image classification, recognition, segmentation, and super-resolution reconstruction. The Inception v3 model [31] in the Google Inception Net [32] series is one of the typical CNN architectures that has been widely used in medical imaging, since it not only performs well on classification results but also keeps a relatively low requirement of calculation and parameters.

**Figure 1:**
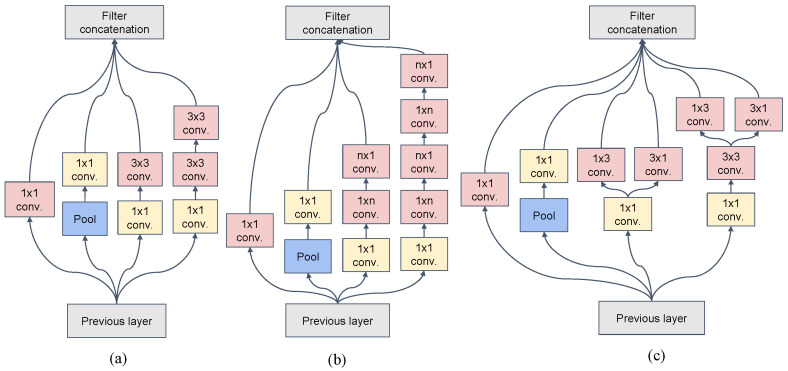
The inception module of Inception v3. (**a**) is the first type of inception module, whose input size is 35 × 35 × 288. (**b**) is the second type of inception module, which splits n × n convolution into n × 1 convolution and 1 × n convolution. (**c**) is the third type of inception module with expanded filter bank outputs.

The backbone components of Inception v3 are shown in Table 2. The character of the network is mainly shown in two aspects. One is to reoptimize the structure of the Inception Module and design three modules, as shown in Figure 1. The other is introducing the idea of factorization into smaller convolutions, which splits a larger convolution into two smaller convolutions. For example, in Figure 1b, the 7 × 7 convolution is divided into 1 × 7 convolution and 7 × 1 convolution. This processing method reduces the parameters and improves the model’s nonlinear expression ability.

### 3.2. Multi-Spectral

Wavelength is a basic attribute of light waves. Only when it ranges about 450–650 nm, the light can be noticed by human eyes and sensed as the feeling of color. The common light is always not mono-wavelength. It is a combination of lights with multiple different wavelengths. If prism or gating is inserted into light path, the light of different wavelengths can be separated from each other. The aim to use multiple narrow wavelength lights is to better investigate target components since different materials have different reflection or transmission capabilities to the light of different wavelengths. So, using multiple mono-wave or narrow wavelength lights as a light source provides more detailed spectral “features” of the target as well as the spatial information, which thus discover more details unseen by traditional RGB images. This is the basic idea of the so-called multi-spectral technology.

Generally, recent multi-spectral imaging technology (number of bands are usually more than 3) can expend from visible to infrared or ultraviolet, and always implemented by alternated layouting multiple filters before image sensor, each of which is designed to allow only the light with specific narrow wavelength to pass and block all other lights.

## 4. Methodology

This paper proposes a vaginitis classification method based on multi-spectral imaging and feature fusion, named MIDV (Multi-spectral Imaging-based Diagnosis for Vaginitis). The main idea of this algorithm is transfer learning and the fusion of a feature layer. In the current diagnosis of vaginitis, RGB images are commonly used. In order to improve the accuracy of classification, we have introduced the technique of multi-spectral imaging. Transfer learning is a solution to build the connection between multi-spectral images and RGB images. Models pre-trained on RGB images can be applied to multi-spectral images through transfer learning, which can make use of existing resources and facilitate the application of new technical means. For multi-spectral image classification, the most common methods based on CNN models mainly include 1D CNN, 2D CNN, and 3D CNN. 1D CNN only uses spectral information, and 2D CNN only uses spatial information. Although 3D CNN can extract both spatial and spectral information, the computational cost of this method is extremely high. To solve this contradiction and realize the joint extraction of spatial and spectral information, we adopt the technique of 2D CNN and spectral feature fusion.

**Figure 2:**
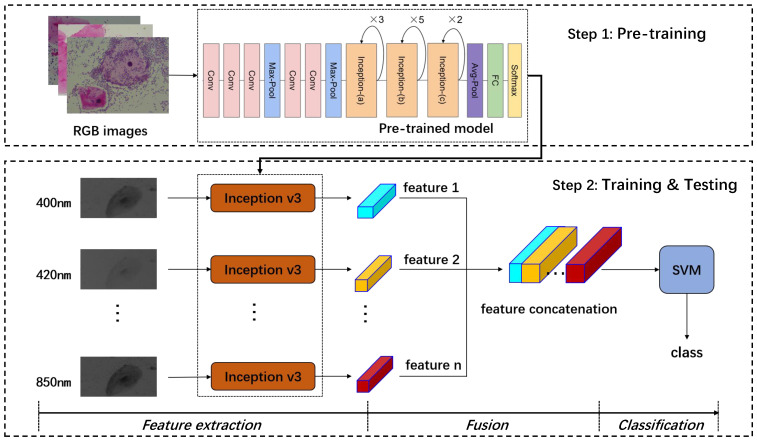
The block diagram of MIDV.

MIDV is shown in Figure 2, which is consisted of two steps: pre-training and then training and testing. The pre-training step uses RGB images to train a standard inception v3 classification model. The training and testing step is comprised of three parts: feature extraction, fusion, and classification. We adopt the transfer learning strategy in the feature extraction part, where the trained inception v3 model in step one is transferred. Although RGB images and multi-spectral images are different images, they are all representations of the same target. RGB images can be seen as rough classified multi-spectrum images, so their image features are related. Therefore, we use this pre-trained model to extract features for each single-spectrum image. In the fusion part, we adopt the strategy of feature layer fusion, as described in Section 2.2. The concatenate method was utilized according to the order of the spectrum from small to large wavelength to take full advantage of the extracted multi-spectral features by the inception v3 model. In the classification part, we use the support vector machine method (SVM) [33] for the reason of simplicity to verify the effect of feature layer fusion.

The specific operation steps of our proposed method are described as follows.

- Step 1. Train an inception v3 model using RGB images of vaginal microorganisms.
- Step 2. The last layer of the inception v3 model as the classifier is removed, so the left parts are used as a feature extractor for multi-spectral images.
- Step 3. Extract features using the inception v3 extractor in Step 2 for every single spectral image in multi-spectral images.
- Step 4. Arrange the features from small to large according to the wavelength of the corresponding single-spectrum image and connect them together with the concatenation operation.
- Step 5. Input the fused feature vector into the SVM classifier, and get the disease category of vaginitis.

## 5. Experiment

### 5.1. Setup

#### 5.1.1. Dataset

The experimental dataset in the present work is comprised of two parts. One is called the primary dataset used to train and test the classifier, and the other is the auxiliary dataset used to pre-train the feature extraction model.

The primary dataset from slide-level labeled is composed of 426,900 multi-spectral images and 426,900 RGB images from 147 patients. In clinical practice, each patient will collect samples on a slide when checking for vaginitis-related diseases, so a slide corresponds to a case. Each patient’s diagnosis result will be marked as the slide-level label by a professional physician. For multi-spectral image collection, we use the specially designed instrument to automatically collect multiple fields of view from one slide and label the images from one slide with the same slide-level annotation. According to the label, there are 11 types of diseases, including normal flora, aerobic vaginitis (AV), bacterial vaginosis (BV), vulvovaginal candidiasis (VVC), flora inhibition, BV + AV mixed infection, BV middle, BV middle + VVC + AV, BV middle + VVC, AV + trichomonas vaginitis (TV), and abnormal flora combined clinically (AFCC). They are the most common diseases in the clinic. The number of images for different diseases is shown in Table 3.

The auxiliary dataset is comprised of RGB images of vaginitis that have different sources as well as fine-grained labels. In clinical use, the laboratory staff collects one or more visual fields that can well support diagnosis results and record them as digital files. So, there are always 1–2 images that are collected for each slide, which can be treated as the very typical example of its disease label. Those images in our auxiliary dataset come from the slide used to construct the primary dataset. However, compared with the random collection, these labels are more accurate and thus can be called typical labels. There are more than 20,000 pictures from more than 20 categories of different diseases. The fine-grained here is the relative concept against the slide-level label.

#### 5.1.2. Image-Collecting Instrument

To collect multi-spectral images, we employ 24 different bandpass filters. Their central wavelength spectrum ranges from 400 to 850 nm. The filter interval is 20 nm, and the half-bandwidth is 14 nm.

### 5.2. Training Strategy

The feature extraction model employs Inception v3. First, vaginitis RGB images in the auxiliary dataset are used to pre-train Inception v3, and the difference is that the classification layer is removed. Then, features are extracted for each spectrum to obtain a 2048-dimensional feature vector. Finally, each spectrum’s feature vectors are combined according to the order of the range, and we input the SVM to get the final classification result. The parameter of SVM is the default value of sklearn.svm.SVC except for the boolean value of probability.

We use the following metrics to compare the classification performance: accuracy, precision, recall, f-score, and kappa value. The higher their values are, the better the classification performance.

### 5.3. Results

#### 5.3.1. Comparison with RGB Image

The multi-spectral image and corresponding RGB images are both grabbed for each field of view under the microscope. Hence, the visible light refers to the RGB image corresponding to the same view area on the scanned glass slide. The comparison with RGB is mainly from two perspectives, which are called multi-classification and binary classification. The former refers to the classification of all 11 disease types in the dataset. The latter refers to each one of the other ten diseases, normal or not. A total of 10 binary classification methods are used to detect the classification effect of a specific vaginitis disease.

**Figure 3:**
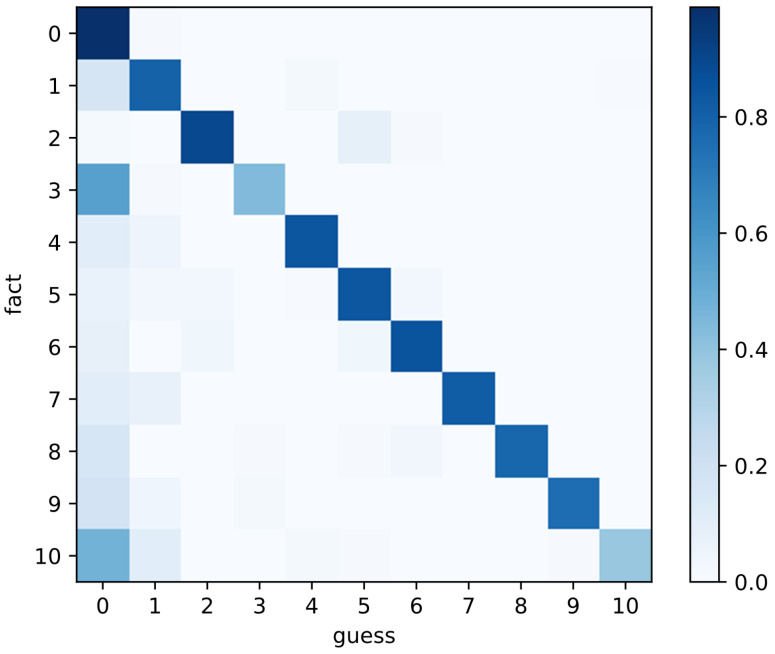
The standard confusion matrix of the MIDV algorithm. 0: normal flora, 1: AV, 2: BV, 3: VVC, 4: flora inhibition, 5: BV + AV, 6: BV middle, 7: BV middle + VVC + AV, 8: BV middle + VVC, 9: AV + TV, 10: abnormal flora combined clinically.

Table 4 presents the five times average value of our proposed multi-spectral image classification algorithm and visible light image classification results. Our algorithm’s classification accuracy rate, precision rate, and recall rate are 11.39%, 15.82%, and 27.25% higher than the RGB image classification results, respectively. Figure 3 is one of the confusion matrixes of MIDV. Since the number of categories is severely unbalanced, we standardize the confusion matrix first and then draw the graph. From the figure, we can see that the classification effect of all diseases is still excellent, which means that our algorithm is not affected by data imbalance.

To thoroughly verify the effectiveness of our proposed algorithm, we also conducted experiments on more CNN-based models, including VGG16 and ResNet50. In this part, the number of epochs used is small to test the effectiveness of the algorithm quickly and keep consistent on test conditions, so the classification accuracy of the Inception v3 model is not as good as the performance shown in Table 4. The experimental results are shown in Table 5. It indicates that multi-spectral images outperform RGB images for all models, and Inception v3 performs better in classification than VGG16 and ResNet50.

**Figure 4:**
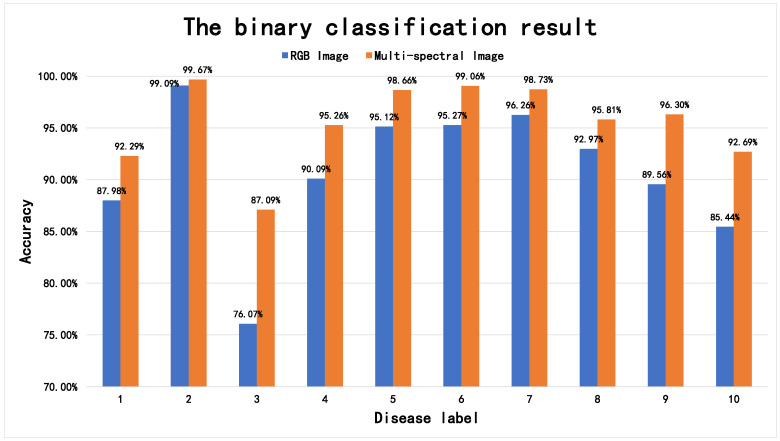
Binary classification results of the multi-spectral image feature fusion and corresponding RGB image classification algorithms. The corresponding relationship between the disease label serial number and the disease category is as follows: 1: AV (VS normal flora), 2: BV (VS normal flora), 3: VVC (VS normal flora), 4: flora inhibition (VS normal flora), 5: BV + AV (VS normal flora), 6: BV middle (VS normal flora), 7: BV middle + VVC + AV (VS normal flora), 8: BV middle + VVC (VS normal flora), 9: AV + TV (VS normal flora), 10: abnormal flora combined clinically (VS normal flora).

Figure 4 shows the binary classification results of the multi-spectral image feature fusion algorithm and corresponding visible light image classification algorithm. The multi-spectral image classification algorithm introduced in this article performs better than the RGB image algorithm for all disease categories. The classification results of diseases such as VVC are most obvious: 11.02% higher.

#### 5.3.2. Comparison with Other Fusion Methods

It has been mentioned in Section 2.2 that standard data fusion methods can be classified as data layer fusion, feature layer fusion, and decision layer fusion. For multi-spectral images, the PCA dimensionality reduction method is usually used in data processing due to the high data dimension. In the present work, the data layer fusion algorithm indicates that the input data is 24 dimensions matching 24 spectra, Then, the 24-dimensional data are transformed into three-dimensional by the PCA method to fit the pre-trained model. Next, the same pre-trained model is applied for feature extraction. Decision layer fusion refers to the feature extraction and classification of the 24 spectrum segments, and the final result is determined by which category appears the most.

Table 6 shows the comparison results of the proposed feature layer fusion algorithm, data layer fusion algorithm, and decision layer fusion algorithm. The performance of feature layer fusion achieves the best result in terms of accuracy, precision, recall, f-score, and kappa. The accuracy value of feature layer fusion is 9.97% higher than data layer fusion and 8.13% higher than decision layer fusion.

Similar to Section 5.3.1, we conduct extended experiments on VGG16 and ResNet50. In this part, the number of epochs used is small, so the classification accuracy of the Inception v3 model is not as good as the performance shown in Table 6. The experimental results are shown in Table 7. The results show that the classification effect of feature layer fusion is better than that of the data layer and decision layer for all CNN-based models. In addition, the feature layer fusion effect of Inception v3 performs best, which indicates that Inception v3 is more effective in extracting multi-spectral image features.

#### 5.3.3. Spectrum Sensitivity

**Figure 5:**
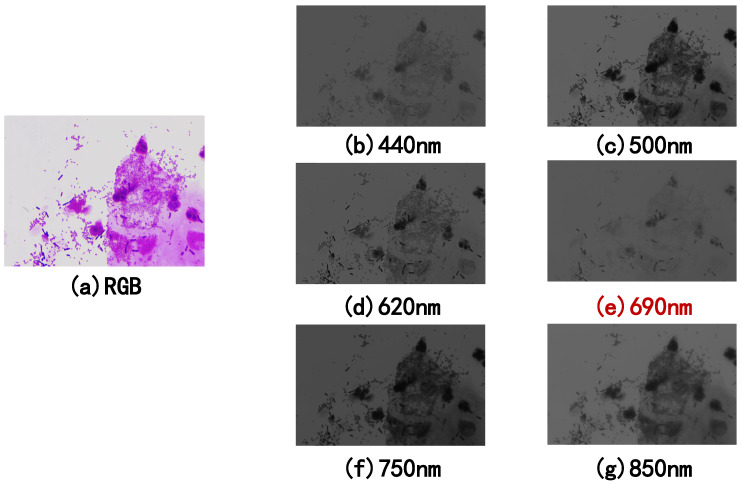
(**a**) is the RGB image of a typical visual field of BV middle + VVC in visible light. (**b**–**g**) are the images collected under the 440 nm, 500 nm, 620 nm, 750 nm, and 850 nm spectrum, respectively. (**e**) is the most sensitive band photograph collected under the 690 nm spectrum. It can be seen that the 690 nm single-spectrum image suppresses some cells and microorganisms in the image while enhancing the lactobacilli compared to the RGB image.

To investigate the impact of each spectrum on the classification results, we also carried out multi-classification and binary classification on each spectrum. Table 8 is the multi-classification result of a single-spectrum image and RGB image. The results show that the best-performing spectrum for the overall accuracy is 600 nm, which is 2.53% higher than the RGB image. For each disease, precision in multi-classification has improved compared with RGB images. We also found that each disease has a feature spectrum. The best performer is BV middle + VVC, where the precision rate increases 31.9% and the corresponding spectrum is 690 nm. A typical visual field of BV middle + VVC is presented in Figure 5. Figure 5a is the RGB image of BV middle + VVC in visible light, and Figure 5e is the most sensitive band photograph collected under the 690 nm spectrum. It can be noticed that the 690 nm spectrum image suppresses some cells and microorganisms in the image and meanwhile enhances the lactobacilli. Table 9 is the comparison result of the binary classification of single-spectrum images and RGB images. The results show that each disease has improved compared with RGB images, and the best-performing spectra are also diverse. The most remarkable improvement is the accuracy of the binary classifications of AV-TV and normal flora.

## 6. Conclusions

This paper introduces multi-spectral images into the auxiliary diagnosis of vaginitis for the first time. It proposes an algorithm based on multi-spectral image feature fusion and transfer learning. Compared with the traditional RGB image classification algorithm, our algorithm has better classification performance in accuracy, precision, recall, f1-score, and kappa value. The classification effect of the Inception v3 model we adopted is also significantly better than that of VGG16 and ResNet50. The feature layer fusion with the 2D CNN method we adopted also performs better than the PCA processing method on the data layer and the element maximum rule method on the decision layer. By using sensitive analysis, we found that each disease has a sensitive band, under which the pathogen is foregrounded and other disruptive components in the image are surpassed. This finding could be used to improve diagnosis algorithms or instruments for vaginitis.

## Figures and Tables

**Table 1:** Recent literature on CAD for vaginitis.

Method Category	Year	References	Approach	Object	Results
SVM	2015	[8]	superpixel and SVM	vaginal bacteria	Accuracy: 89.27%
	2016	[9]	CNN and SVM	candida	Recall: 72%
deep learning	2008	[10]	BP neural network	white blood cells (5 types)	Accuracy: 83.7%
	2018	[11]	deep residual learning theory	white blood cells (40 types)	Accuracy: 76.84%
	2019	[12]	Faster R-CNN	white blood cells (8 types)	Precision:69.94% Recall: 74.15% mAP: 61.74%
	2020	[13]	CNN	bacterial vaginosis (3 types)	Accuracy: 75.1%
laws texture energy	2015	[14]	laws texture energy and threshold segmentation	lactobacilli	Accuracy: 94.2%
	2016	[15]	CNN and SVM	cue cells, epithelial cells	Accuracy: 90.07%

**Table 2:** The network architecture of Inception v3.

Components	Patch Size/Stride or Remarks	Input Size
conv	3 × 3/2	299 × 299 × 3
conv	3 × 3/1	149 × 149 × 32
conv padded	3 × 3/1	147 × 147 × 32
pool	3 × 3/2	147 × 147 × 64
conv	3 × 3/1	73 × 73 × 64
conv	3 × 3/2	71 × 71 × 80
conv	3 × 3/1	35 × 35 × 192
3 × Inception	As in Figure 1a	35 × 35 × 288
5 × Inception	As in Figure 1b	17 × 17 × 768
2 × Inception	As in Figure 1c	8 × 8 × 1280
pool	8 × 8	8 × 8 × 2048
linear	logits	1 × 1 × 2048
softmax	classifier	1 × 1 × 1000

**Table 3:** The primary dataset.

Label_Index	Label_Name	Image_Count	Percentage
0	normal flora	228,275	53.47%
1	AV	48,075	11.26%
2	BV	25,600	6.00%
3	VVC	30,300	7.10%
4	flora inhibition	26,950	6.31%
5	BV + AV	29,950	7.02%
6	BV middle	11,775	2.76%
7	BV middle + VVC + AV	5450	1.28%
8	BV middle + VVC	7275	1.70%
9	AV + TV	3750	0.88%
10	AFCC	9500	2.23%
all	sum	426,900	100.00%

**Table 4:** Comparison with RGB Image (multiple-label classification).

Data_Type	Accuracy	Precision	Recall	F1-Score	Kappa
RGB image	76.04%	77.36%	48.35%	53.69%	61.07%
multi-spectral image (ours)	87.43%	93.18%	75.60%	81.66%	80.42%

**Table 5:** Comparison with RGB Image in more CNN-based models (multiple-label classification).

Model	Data_Type	Accuracy	Precision	Recall	F1-Score	Kappa
VGG16	RGB image	61.99%	42.51%	32.78%	30.30%	38.82%
	multi-spectral image	66.56%	77.49%	58.14%	62.89%	50.97%
ResNet50	RGB image	72.68%	59.98%	46.77%	49.12%	56.14%
	multi-spectral image	77.28%	84.72%	71.18%	72.56%	64.97%
Inception v3	RGB image	68.90%	52.49%	41.86%	42.36%	49.48%
	multi-spectral image (fewer epochs)	84.65%	85.97%	85.25%	83.11%	78.30%

**Table 6:** Comparison of other fusion methods.

Fusion Type	Accuracy	Precision	Recall	F1-Score	Kappa
data layer fusion	77.46%	80.23%	53.78%	59.57%	63.56%
decision layer fusion	79.30%	89.32%	55.49%	62.67%	65.95%
feature layer fusion (ours)	87.43%	93.18%	75.60%	81.66%	80.42%

**Table 7:** Comparison of other fusion methods on more CNN-based models.

Model	Fusion Type	Accuracy	Precision	Recall	F1-Score	Kappa
VGG16	data layer fusion	52.41%	6.49%	9.61%	7.36%	1.22%
	decision layer fusion	53.52%	11.18%	9.16%	6.47%	0.21%
	feature layer fusion	66.56%	77.49%	58.14%	62.89%	50.97%
ResNet50	data layer fusion	53.51%	8.65%	9.15%	6.45%	0.16%
	decision layer fusion	55.16%	18.50%	11.46%	10.29%	6.03%
	feature layer fusion	77.28%	84.72%	71.18%	72.56%	64.97%
Inception v3	data layer fusion	52.90%	16.66%	17.39%	14.77%	20.78%
	decision layer fusion	57.19%	41.70%	17.46%	19.04%	11.65%
	feature layer fusion(fewer epochs)	84.65%	85.97%	85.25%	83.11%	78.30%

**Table 8:** Multi-classification result of single-spectrum image and RGB image, and AFCC is abnormal flora combined clinically.

Label_Name	Most Sensitive Spectrum	Single-Spectrum Precision	RGB Precision	Improve
accuracy	600 nm	78.57%	76.04%	2.53%
normal flora	640 nm	78.90%	77.10%	1.80%
AV	540 nm	73.60%	64.90%	8.70%
BV	850 nm	87.50%	82.90%	4.60%
VVC	580 nm	95.70%	85.60%	10.10%
BV-AV	810 nm	84.70%	75.40%	9.30%
BV middle	850 nm	79.20%	62.30%	16.90%
BV middle + VVC + AV	580 nm	95.80%	84.40%	11.40%
BV middle + VVC	690 nm	92.30%	60.40%	31.90%
AV-TV	480 nm	100.00%	84.80%	15.20%
AFCC	480 nm	100.00%	90.00%	10.00%

**Table 9:** Binary classification result of single-spectrum image and RGB image, and AFCC is abnormal flora combined clinically.

Label_Name	Most Sensitive Spectrum	Single-Spectrum Precision	RGB Precision	Improve
AV-TV	670 nm	96.22%	89.56%	6.67%
VVC	750 nm	80.21%	76.07%	4.13%
BV middle	640 nm	98.09%	95.27%	2.83%
AFCC	580 nm	88.16%	85.44%	2.72%
flora inhibition	650 nm	92.41%	90.09%	2.32%
BV-AV	810 nm	97.23%	95.12%	2.11%
BV middle + VVC	440 nm	94.63%	92.97%	1.66%
BV middle + VVC + AV	730 nm	97.86%	96.26%	1.60%
AV	540 nm	89.19%	87.98%	1.20%
BV	460 nm	99.46%	99.09%	0.37%

## Data Availability

The data presented in this study are available on request from the corresponding author.

## References

[1] (2020). China Health Statistical Year Book.

[2] Shen Y., Bao J., Tang J., Liao Y., Liu Z., Liang Y. (2019). Relationship between vaginitis, HPV infection and cervical cancer. Chin. J. Women Child. Health.

[3] Meng L., Xue Y., Yue T., Yang L., Gao L., An R. (2016). Relationship of HPV infection and BV, VVC, TV: A clinical study based on 1261 cases of gynecologic outpatients. Chin. J. Obstet. Gynecol..

[4] Yang X., Luo W., Xing L., Liu D., He R. (2019). Research progress on the relationship between candidal vaginitis during pregnancy and adverse pregnancy outcome. Chin. J. Mycol..

[5] Ding H., Cao D. (2007). Analysis of related factors of misdiagnosis of vaginitis secretion under microscope. Chin. J. Clin. Lab. Sci..

[6] Zhu A. (2009). Analysis of related factors affecting the results of vaginal secretion examination. Guide China Med..

[7] Li Y., Feng W., Wang P., Xu Z., Bai Q., Liu C. (2014). Analysis of related factors of misdiagnosis of bacterial vaginosis secretions. J. Aerosp. Med..

[8] Song Y., Lei B., He L., Zeng Z., Zhou Y., Ni D., Chen S., Wang T. (2015). Automatic Detection of Vaginal Bacteria Based on Superpixel and Support Vector Machine. Chin. J. Biomed. Eng..

[9] Guo R. (2016). Pattern Recognition Research of Microscope Wet Leucorrhea Image Based on CNN-SVM. Master’s Thesis.

[10] Zhang L. (2008). The Classification Research on Microscopic Leucocyte Image. Master’s Thesis.

[11] Qin F., Gao N., Peng Y., Wu Z., Shen S., Grudtsin A. (2018). Fine-grained leukocyte classification with deep residual learning for microscopic images. Comput. Methods Programs Biomed..

[12] Yan S. (2019). Research on the Effect of Generative Adversarial Network on Improving the Accuracy of Leucorrhea Microscopic Image Detection. Master’s Thesis.

[13] Wang Z., Zhang L., Zhao M., Wang Y., Bai H., Wang Y., Rui C., Fan C., Li J., Li N. (2020). Deep neural networks offer morphologic classification and diagnosis of bacterial vaginosis. J. Clin. Microbiol..

[14] Guo Y., Ma L., Li J. (2015). Detection and Statistical Analysis of Lactobacillus in Gynecological Medical Micrographs. J. Data Acquis. Process..

[15] Ma L. (2016). Components Statistics and Analysis Based on Texture Analysis in Gynecologic Microscopic Image. Master’s Thesis.

[16] Yan J., Chen H., Liu L. (2019). Overview of hyperspectral image classification. Opt. Precis. Eng..

[17] Liu W., Wang L., Liu J., Yuan J., Chen J., Wu H., Xiang Q., Yang G., Li Y. (2016). A comparative performance analysis of multispectral and rgb imaging on her2 status evaluation for the prediction of breast cancer prognosis. Transl. Oncol..

[18] Liu W.L., Wang L.W., Chen J.M., Yuan J.P., Xiang Q.M., Yang G.F., Qu A.P., Liu J., Li Y. (2016). Application of multispectral imaging in quantitative immunohistochemistry study of breast cancer: A comparative study. Tumor Biol..

[19] Qi X., Xing F., Foran D.J., Yang L. (2011). Comparative performance analysis of stained histopathology specimens using RGB and multispectral imaging. Medical Imaging 2011: Computer-Aided Diagnosis.

[20] Pan S.J., Yang Q. (2009). A survey on transfer learning. IEEE Trans. Knowl. Data Eng..

[21] Maghdid H.S., Asaad A.T., Ghafoor K.Z., Sadiq A.S., Mirjalili S., Khan M.K. (2021). Diagnosing COVID-19 pneumonia from X-ray and CT images using deep learning and transfer learning algorithms. Multimodal Image Exploitation and Learning 2021.

[22] Liu W., Cheng Y., Liu Z., Liu C., Cattell R., Xie X., Wang Y., Yang X., Ye W., Liang C. (2021). Preoperative prediction of Ki-67 status in breast cancer with multiparametric MRI using transfer learning. Acad. Radiol..

[23] Esteva A., Kuprel B., Novoa R.A., Ko J., Swetter S.M., Blau H.M., Thrun S. (2017). Dermatologist-level classification of skin cancer with deep neural networks. Nature.

[24] Wahab N., Khan A., Lee Y.S. (2019). Transfer learning based deep CNN for segmentation and detection of mitoses in breast cancer histopathological images. Microscopy.

[25] Hall D., Llinas J. (2001). Multisensor Data Fusion.

[26] Zhang H., Cheng C., Xu Z., Li J. (2020). Survey of data fusion based on deep learning. Comput. Eng. Appl..

[27] Meng T., Jing X., Yan Z., Pedrycz W. (2020). A survey on machine learning for data fusion. Inf. Fusion.

[28] Liu Y., Zhang C., Cheng J., Chen X., Wang Z.J. (2019). A multi-scale data fusion framework for bone age assessment with convolutional neural networks. Comput. Biol. Med..

[29] Zhang L., Gao H., Wen J., Li S., Liu Q. (2017). A deep learning-based recognition method for degradation monitoring of ball screw with multi-sensor data fusion. Microelectron. Reliab..

[30] Fu H., Ma H., Wang G., Zhang X., Zhang Y. (2020). MCFF-CNN: Multiscale comprehensive feature fusion convolutional neural network for vehicle color recognition based on residual learning. Neurocomputing.

[31] Szegedy C., Vanhoucke V., Ioffe S., Shlens J., Wojna Z. Rethinking the inception architecture for computer vision. Proceedings of the IEEE Conference on Computer Vision and Pattern Recognition.

[32] Szegedy C., Liu W., Jia Y., Sermanet P., Reed S., Anguelov D., Erhan D., Vanhoucke V., Rabinovich A. Going deeper with convolutions. Proceedings of the IEEE Conference on Computer Vision and Pattern Recognition.

[33] Hearst M.A., Dumais S.T., Osuna E., Platt J., Scholkopf B. (1998). Support vector machines. IEEE Intell. Syst. Their Appl..

